# The Transformative Potential of Lipid Nanoparticle–Protein Corona for Next-Generation Vaccines and Therapeutics

**DOI:** 10.1021/acs.molpharmaceut.3c00479

**Published:** 2023-10-02

**Authors:** Augusto Amici, Daniela Pozzi, Cristina Marchini, Giulio Caracciolo

**Affiliations:** † School of Biosciences and Veterinary Medicine, University of Camerino, Via Gentile III da Varano, 62032 Camerino, Italy; ‡ NanoDelivery Lab, Department of Molecular Medicine, Sapienza University of Rome, Viale Regina Elena 291, 00161 Rome, Italy

**Keywords:** SARS-CoV-2, vaccination, lipid nanoparticle, protein corona

## Abstract

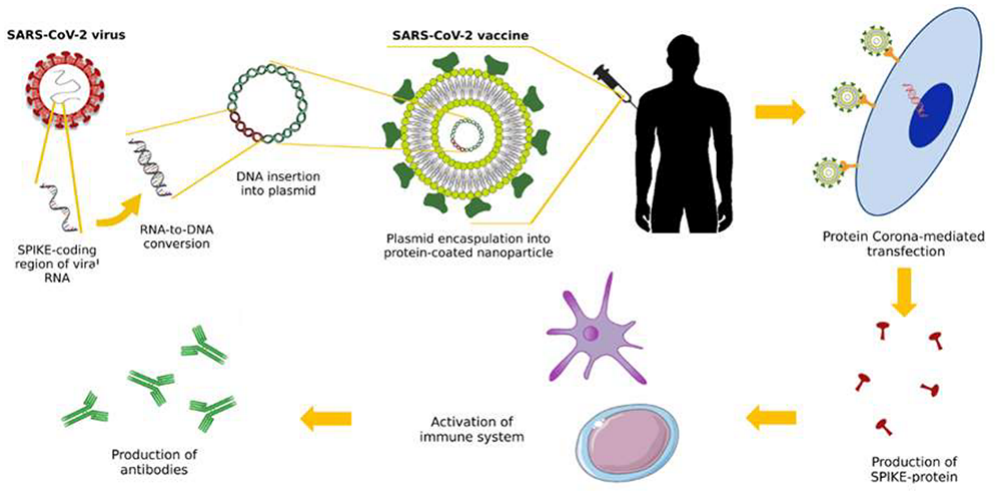

The integration of the lipid nanoparticle (LNP)-protein corona as a pioneering approach for the development of vaccines against the present and future SARS-CoV-2 variants of concern marks a significant shift in the field. This concept holds great promise, offering a universal platform that can be adaptable to combat future pandemics caused by unknown viruses. Understanding the complex interactions among the protein corona, LNPs, and receptors is crucial for harnessing its potential. This knowledge will allow optimal vaccine formulations and improve their effectiveness. Safety assessments are essential to ensure suitability for human use, compliance with regulatory standards, and rigorous quality control in manufacturing. This transformative workflow requires collaborative efforts, expanding our foundational knowledge and translating advancements from the laboratory to clinical reality. The LNP-protein corona approach represents a paradigmatic shift with far-reaching implications. Its principles and insights can be leveraged beyond specific applications against SARS-CoV-2, enabling a universal platform for addressing viral threats, cancer, and genetic diseases.

To date, the most promising vaccines in terms of efficacy against symptomatic COVID-19 infection are the genetic vaccines containing mRNA (mRNA) encapsulated in lipid nanoparticles (LNPs).^1^ Among these vaccines, two notable examples are mRNA-1273/SpikeVax, developed by Moderna, and BNT162b2/Comirnaty, developed by BioNTech/Pfizer. These ground-breaking vaccines have demonstrated significant effectiveness in combating the spread of COVID-19 and have played a crucial role in global vaccination efforts. By utilizing mRNA technology and LNPs as delivery systems, these vaccines have shown encouraging results in stimulating immune responses and reducing the severity of the disease. Their development and subsequent authorization for emergency use represent significant milestones in the fight against the COVID-19 pandemic.

In June 2020, the pressure exerted by the pandemic forced the European Commission to propose an EU vaccine strategy to expedite the development, production, and deployment of COVID-19 vaccines, wherein research and innovation play a vital role. Moderna and BioNTech/Pfizer turned to LNP delivery systems at the beginning of 2020. However, the ground-breaking potential of LNPs as a delivery system for RNA-based candidates was demonstrated earlier by Onpattro, a prescription medication that received FDA approval in 2018 for the treatment of polyneuropathy associated with hereditary transthyretin-mediated amyloidosis (hATTR amyloidosis).^2^ This research paved the way for multiple clinical translations of RNA-based therapies.^3^ From the very beginning of this exciting new era, numerous patent wars have broken out, with Alnylam accusing Pfizer and Moderna of violating its delivery technology in their COVID-19 vaccines.^4^ Alnylam claims that four patents were infringed by Pfizer and three by Moderna. Moderna countersued, alleging baseless profit-seeking by Alnylam from its inventions. Other patent suits involve Arbutus Biopharma and Genevant Sciences accusing Moderna of infringing six patents and CureVac accusing Pfizer and BioNTech of violating three patents. Pfizer dismisses these claims as profit-seeking attempts. Moderna and Pfizer are in a court battle, with mutual accusations escalating and Pfizer countering. This perspective abstains from making any evaluative judgments, but the authors cannot disregard the colossal commercial ramifications of the COVID-19 vaccination campaigns.

Reverting to scientifically relevant topics, several studies have shown that LNP vaccines have significantly contributed to preventing severe outcomes, including hospitalization and mortality,^5^ and the spread of novel variants.^6^ On the other hand, it is important to acknowledge that these vaccines are not without limitations. As with any medical intervention, there is always room for optimization and improvement.^7^ To ensure widespread distribution, there is a need for vaccine formats that have lower barriers, in terms of manufacturing, distribution, and administration. Overcoming these technological hurdles is crucial to achieving global vaccine equity and ensuring that populations in low-income countries have access to effective COVID-19 vaccines. Figure 1 highlights some of the key issues discussed in this Perspective.

**Figure 1:**
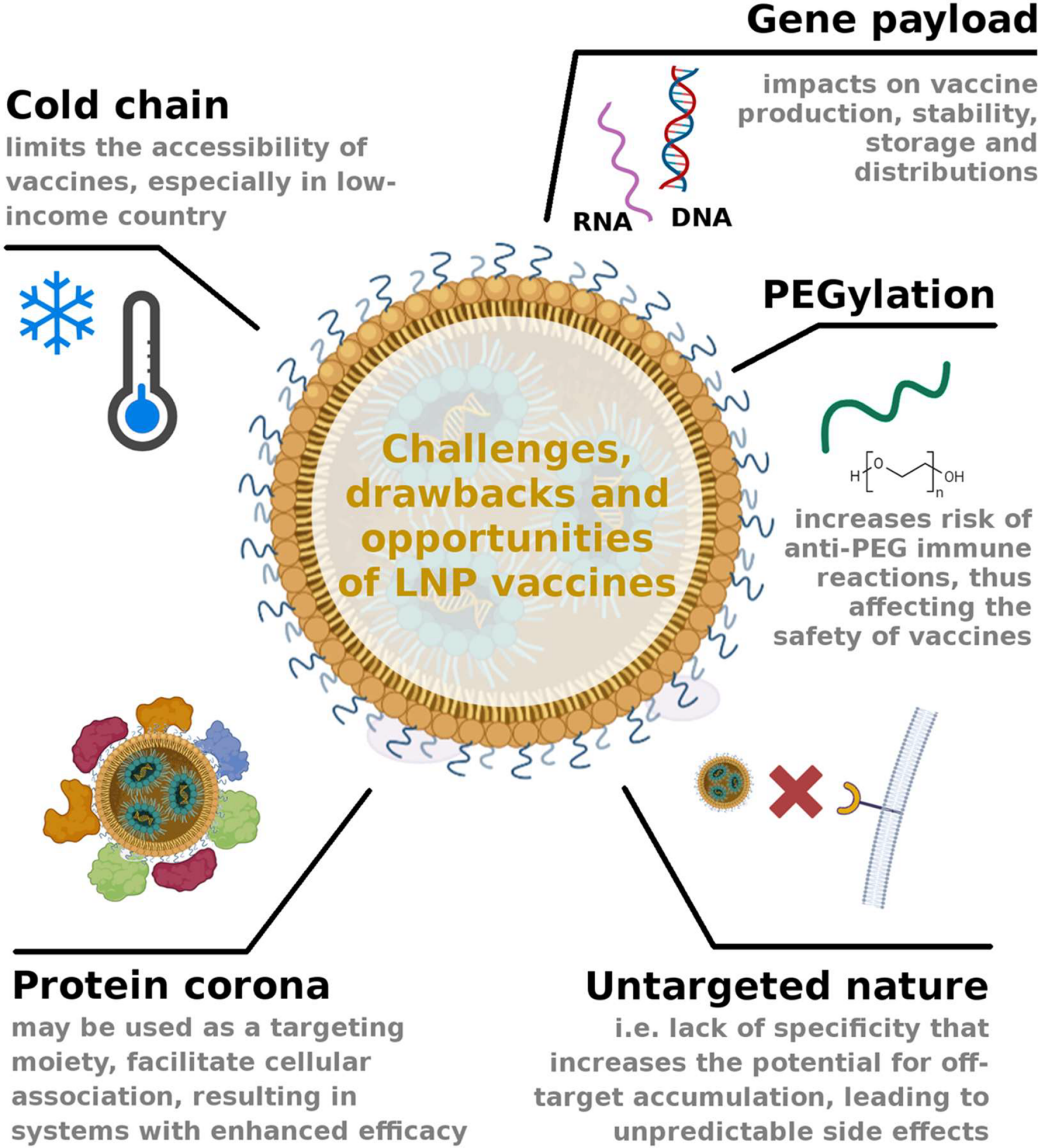
Cartoon underscores the primary challenges and drawbacks associated with LNP vaccines, while also highlighting potential opportunities. One significant opportunity lies in the ability to modify the protein corona composition, which can help mitigate off-target accumulation and enhance the interaction of LNPs with antigen-presenting cells and dendritic cells. This, in turn, has the potential to significantly improve vaccine efficacy.

**Developing a thermostable LNP vaccine** presents a significant technological challenge with the goal of preserving RNA integrity and vaccine effectiveness. All currently authorized RNA vaccines available in the United States still require **cold chain storage**.^8^ However, the storage requirements for approved vaccines are far from uniform and each vaccine comes with its own specific storage demands.^8^ For instance, BNT162b2/Comirnaty demands an ultracold environment, chilling down to −80 °C with a shelf life of up to 6 months, while the mRNA-1273/SpikeVax requires a comparably milder storage temperature of −20 °C yet still sustains the same extended shelf life. The variation in storage conditions may be due to additional precautions taken by BioNTech/Pfizer. According to this, stability study data submitted by Pfizer to the European Medicines Agency (EMA) indicates that BioNTech/Pfizer vaccines have a similar shelf life (30 days) to Moderna’s vaccine when refrigerated at 2–8 °C.^8^ However, given the well-known impact of lipid composition on the thermotropic behavior of lipid mixtures,^9^ it is reasonable to consider that the stability of a vaccine formulation could be affected by its lipid composition across different temperature conditions. Further research and analysis are warranted to unravel the intricate relationship between lipid components and the observed differences in thermostability between Moderna’s vaccine and others, providing valuable insights for future vaccine design and optimization.

In any case, the requirement for cold chain storage and distribution poses challenges to the accessibility of vaccines, particularly in low-income countries with limited infrastructure to maintain the cold chain. To address this challenge, various alternatives have been suggested. Notably, efficient drying technologies that eliminate water from vaccine formulations have been shown to extend vaccine stability, both at room temperature and in refrigerated conditions.^10,11^ Two widely used drying methods are freeze-drying (lyophilization) and spray drying. Freeze-drying, a well-established technique for preserving biologics, such as vaccines, entails freezing the formulation to create a solid matrix. Water is then removed through sublimation under precise conditions, resulting in a stable, dried product. Lyophilization, which requires specialized equipment and controlled environments, ensures the integrity of RNA.

Recently, Meulewaeter and colleagues employed a freeze-drying method based on spin-freezing to produce optimally lyophilized mRNA LNPs that can be safely stored at higher temperatures for months without losing their transfection properties.^12^

Alternatively, spray drying atomizes the vaccine formulation into tiny droplets that are dried rapidly with hot air. Fine-tuning parameters such as temperature, pressure, and drying time are crucial to ensure RNA vaccine stability. While spray drying can suit RNA vaccines, it may necessitate the use of stabilizers and optimization to prevent RNA degradation. Selecting suitable protectants or stabilizers is pivotal to shielding RNA from harm during drying and subsequent rehydration.

The initial stride toward augmenting the accessibility of LNP vaccines entails the utilization of plasmid DNA to encode protein antigens, as opposed to mRNA. Employing DNA technology presents several advantages over mRNA-based vaccines.^13^ Among them, DNA vaccines demonstrate enhanced stability and obviate the need for a cold chain infrastructure during storage and transportation.

Another drawback is that **the approved LNPs are PEGylated**, which means they have poly(ethylene glycol) (PEG) molecules attached to their surface. For a long time, PEG has been widely used as a preferred strategy to confer stealth properties to systemically administered NPs to prolong circulation time and biodistribution.^14^ Concerns regarding the applicability of PEGylation due to potential anti-PEG immune reactions have been well-known for many years,^15^ which may pose risks and affect the safety and efficacy of the vaccine.^16^ Recent review papers provide a focused examination of the hypersensitivity reactions attributed to the use of PEGylated products, specifically PEG-based mRNA COVID-19 vaccines^17^ and explores potential alternatives to PEGylation, aiming to improve vaccine safety and reduce unwanted immune responses. For instance, utilizing short-chain PEG could be a potential option to minimize the immune response and the formation of anti-PEG antibodies. However, further studies and careful consideration of specific applications are necessary to fully evaluate the benefits and potential drawbacks of employing short-chain PEG as an alternative.

Nevertheless, in situations where LNP vaccines are intended for localized administration, like intradermal delivery (ID),^18^ and their interaction with plasma proteins is limited compared to systemic circulation, employing unPEGylated materials may counteract the observed increase in anti-PEG antibodies.^19^ ID is a convenient administration route as it stimulates robust immune responses for vaccination due to the higher concentration of antigen-presenting cells (APCs) in the dermis and fewer systemic adverse effects.^20^

However, it should be underlined that one of the main challenges in using LNPs as drug delivery vehicles is their tendency to aggregate. By coating LNPs with PEG, the hydrophilic PEG chains create a steric hindrance that hinders the interaction of LNPs with each other, thus preventing aggregation. Hence, if effective strategies are developed to produce stealth LNPs without relying on PEGylation, it will also be essential to devise methods to reduce their tendency to aggregate.

Another constraint of the approved LNPs is their **untargeted nature**, resulting in a lack of specificity regarding their delivery to cells or tissues. This lack of targeting capability increases the potential for unwanted, off-target accumulation of the vaccine components, which can potentially lead to unpredictable side effects. LNP optimization for the efficient transfection of APCs, having a crucial role in the immune response, might improve the efficiency of the current mRNA vaccines.

One of the primary goals of future research will be to enhance LNP vaccines with a distinct targeting ability, specifically directed toward APCs. Among possible targeting strategies (reviewed in ref (21)), one of the most consolidated approaches involves the conjugation of ligands to PEG moieties, creating stealth and targeted nanomedicine. This strategy aims to combine the supposed benefits of PEGylation with the specific binding capabilities of ligands, enabling targeted delivery to specific cells or tissues. However, issues related to ligand specificity, stability, and binding affinity play crucial roles in the success of targeted delivery. Other challenges include the heterogeneity and dynamic nature of target cells, which can exhibit variations in receptor expression and internalization mechanisms. Furthermore, the immune system’s response to targeted NPs, such as the development of antidrug antibodies or immune clearance, poses additional hurdles to successful delivery. Despite extensive research in this field, the translation of targeted NP delivery into clinical applications has been therefore hindered, resulting in limited success.^22^ In recent times, novel opportunities have emerged in the realm of bionano interactions, presenting new avenues for the development of targeted NPs. These interactions result in the encapsulation of NPs by biomolecules, leading to the formation of a biomolecular corona, commonly known as the protein corona, due to its predominant enrichment with plasma proteins. Initially, it was observed that the presence of a protein corona on NPs hinders the effectiveness of targeting ligands,^23^ thereby impairing the NP’s targeting ability. However, it was later recognized that complete avoidance of protein corona formation, even in PEGylated systems, is not feasible.^24^ Consequently, efforts were redirected toward **harnessing the protein corona as an inherent targeting moiety**, as the proteins acquired from the bloodstream could potentially serve as targeting ligands.^25^

Our research group has successfully provided direct evidence of targeted delivery of NPs by coating a cationic liposome with a protein corona enriched in vitronectin.^26^ This plasma protein bestowed the liposome with the remarkable ability to selectively target and bind to cancer cells that exhibit an elevated level of expression of the vitronectin receptor. A recent scientific hypothesis has suggested that the high liver tropism and transfection potency exhibited by Onpattro may be attributed to the formation of a protein corona enriched with Apo-E.^2^ This protein corona, in turn, enables the recognition of hepatocytes through the LDL receptor, potentially contributing to its liver-targeting capabilities.

In the context of vaccination, the translation of LNP vaccines into clinical practice faces challenges due to our limited understanding of their protein corona, which may influence the tissue accumulation of particles in physiological environments. Upon ID, LNP vaccines are exposed to the dermal interstitial fluid (DIF)^27^ leading to the formation of a protein corona. Given that the protein source is a key determinant in shaping the composition of the protein corona,^28^ we assert that conducting a thorough investigation of LNPs in the DIF is an essential and decisive step in advancing our comprehension of their mechanism of action upon intradermal injection. By rigorously examining the behavior and interactions of LNPs within this specific environment, we can gain crucial insights into their functional attributes, including their stability, targeting efficiency, and immunogenicity. This in-depth investigation will provide valuable knowledge to further optimize LNP-based therapeutics and foster the development of innovative strategies for the effective delivery and enhanced therapeutic outcomes in dermal applications. An optimized protein corona may facilitate cellular association and activate signaling pathways, leading to enhanced immune responses (Figure 2). Although extensive research efforts have been dedicated to investigating the interactions between NPs and cellular receptors,^29^ the intricate engagement at the interface between NPs and receptors has not been fully elucidated and needs new efforts and technologies.^30^ However, factors such as shear stress, which plays a role in shaping the protein corona,^31^ are difficult to replicate accurately in vitro. Consequently, the development of sophisticated in vitro models that faithfully capture the intricate interplay between NPs and the surrounding biological milieu^32^ stands as a crucial objective to optimize vaccination strategies based on the exploitation of the protein corona.

**Figure 2:**
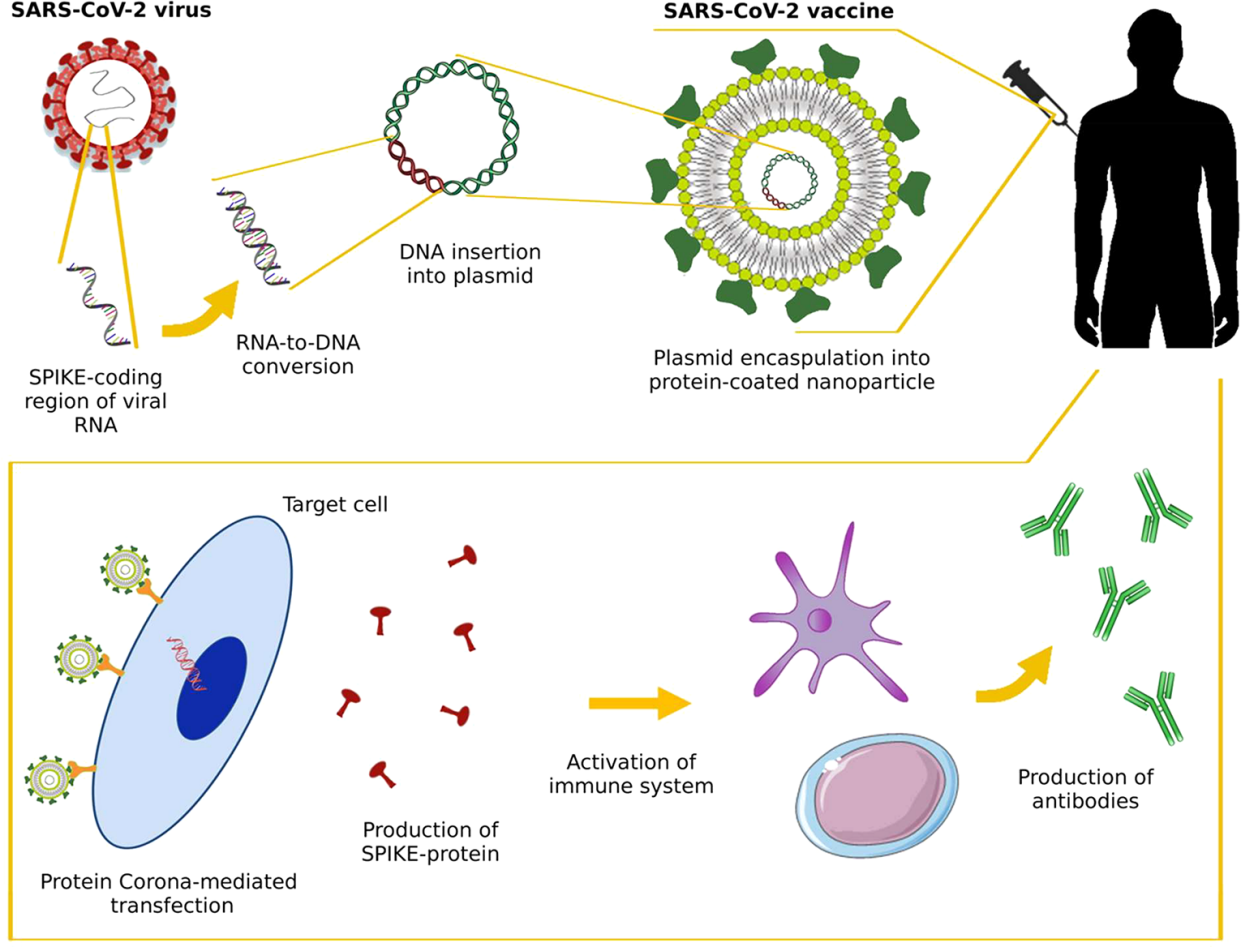
DNA vaccines targeting SARS-CoV-2 can be created through recombinant DNA technology, utilizing the commercially available pcDNA3.1-SARS2-Spike plasmid (e.g., obtained from Addgene, MA) as the template. A DNA plasmid encoding the extracellular domain of the spike protein is loaded into a lipid nanoparticle (LNP). Next, the production of LNP-protein corona DNA vaccines will follow, achieved by incubating DNA-loaded LNPs with donor-derived interstitial fluid (DIF) obtained from healthy human volunteers, employing standardized protocols. The protein corona will consist of numerous proteins, here represented by a single green protein for simplicity. Our focus will shift toward exploiting the protein corona as a natural targeting entity, with particular attention to certain proteins, known as protein corona fingerprints (PCFs), which hold promise as potential targeting ligands. The internalization of LNP-protein corona DNA vaccines into target cells, such as antigen-presenting cells (APCs) and dendritic cells (DCs), ensues via a receptor-mediated mechanism. Protein corona fingerprints lock onto receptors of APCs stimulating massive vaccine internalization, which then churns out copies of the virus’s spike protein. Activation of adaptive immunity leads to the production of neutralizing antibodies and cell-mediated immune responses against SARS-CoV-2. For simplicity, the cell-mediated immune response is not depicted in the figure, but for a complete description, the reader may refer to.^39^

To ensure the efficacy and safety of nanoenabled vaccination utilizing the protein corona, it is essential to implement a comprehensive workflow that encompasses multiple stages, ranging from experimental design to rigorous clinical investigations. This workflow aims to ensure a rigorous and systematic approach in the development and evaluation of protein corona-based vaccines.

The initial step involves designing well-structured experiments to systematically investigate the interaction between NPs and biomolecules, leading to the formation of the protein corona. Design of Experiments (DoE) techniques can be employed to efficiently screen and optimize key parameters, such as NP composition, surface modifications, and protein corona formation conditions.^33^ DoE enables the simultaneous variation of multiple factors to identify the optimal configuration of parameters that maximize desired outcomes while minimizing the number of experimental runs required. So far, the DoE has primarily been employed to reduce the number of experiments in in vitro studies. However, if applied to in vivo investigations, it has the potential to unveil unforeseen correlations that might catalyze further research into the underlying mechanisms responsible for these correlations.

A library of DNA-loaded LNPs with diverse synthetic identities should be generated by utilizing microfluidic platforms. Subsequently, LNP-protein corona DNA vaccines can be produced by incubating DNA-loaded LNPs with DIF obtained from healthy human volunteers through standardized procedures. LNP-protein corona vaccines will vary in their lipid composition, lipid/DNA molar ratio, and lipid/DIF volume ratio. Multiple synthetic properties will be measured as Xn variables. Furthermore, various in vitro and in vivo outcomes such as cellular uptake, transfection efficiency, cellular cytotoxicity at different time points, dendritic cell (DC) maturation (both phenotype and function), biodistribution, global cytokine profile, and induction of neutralizing antibodies in vaccinated mice will be evaluated as Yn variables. Quantitative structure–activity relationship (QSAR) methods will be used to clarify the correlations between the descriptors and the measured biological end points to model *y* as a function of *x*, *y* = *f*(*x*).^34,35^ By harnessing QSAR techniques, we can accelerate the screening and optimization of LNP-protein corona DNA vaccines, ultimately facilitating the design of more effective and targeted vaccine delivery systems.^34^

Subsequently, to ensure the effectiveness and safety of LNP-protein corona DNA vaccines, several important steps must be taken. Next, the ability of the most efficient LNP-protein corona DNA vaccines to induce antiviral immunity will be thoroughly investigated. Additionally, extensive pharmacological and toxicological studies will be conducted to evaluate the safety and efficacy profiles of the LNP-protein corona DNA vaccines. These studies are essential for understanding any potential adverse effects and ensuring the vaccine’s overall safety in human subjects. Recent research has demonstrated that grafting the protein corona to LNPs eliminates gender bias, making it a robust strategy for developing a delivery platform that can produce therapeutic effects independent of the patient’s sex.^36^ This finding highlights the potential advantage of utilizing the protein corona for the targeted delivery of LNP-based vaccines, as it may equally benefit both men and women. By rigorously investigating stability, immune response, safety, and regulatory requirements, LNP-protein corona DNA vaccines can advance toward clinical trials, offering a promising strategy for effective and unbiased vaccination against COVID-19 and potentially other viral diseases. One important consideration regarding the production of thermostable LNP-protein corona DNA vaccines using drying methods is that these methods can affect proteins in various ways, potentially causing denaturation, aggregation, and oxidative damage.^37^ Denaturation alters the protein structure, reducing function. Aggregation can occur, especially in concentrated dry matrices, which diminishes protein effectiveness. Exposure to oxygen during drying can lead to oxidative damage, altering the protein structure and function. Some drying methods such as spray drying or freeze-drying may harm protein activity due to structural changes. To mitigate these issues and protect proteins during the drying process, researchers should use stabilizers under optimized drying conditions (temperature, humidity, and drying rate).

When a risk assessment of DNA vaccines is conducted, several key factors must be taken into consideration. These factors include biodistribution, the persistence and integration of plasmid DNA into the chromosome, local reactogenicity, systemic toxicity, undesirable biological activity, and the potential for autoimmunity. It is worth noting that extensive evidence indicates that plasmid DNA is rapidly eliminated from the body, and delivery methods such as intramuscular, subcutaneous, intradermal, or particle-mediated approaches do not result in long-term persistence of DNA plasmids at unintended sites or their integration into the host genome.^38^ The approval of ZyCoV-D, a DNA vaccine developed against SARS-CoV-2 by Cadila and Zydus, is a significant milestone for DNA vaccines. It can serve as a catalyst for the development of other DNA-based vaccines that target various infectious diseases or cancer. ZyCoV-D has demonstrated full protection against severe disease and death, while remaining safe and stable at room temperature. However, it is important to note that ZyCoV-D requires a physical method of delivery, specifically a needle-free injection system, which may pose a logistical challenge for widespread use. LNP-protein corona DNA vaccines might overcome these delivery system challenges, offering a solution for a global DNA vaccine application. The forthcoming research endeavors will be dedicated to an intricate pursuit: the quest to discern an exquisitely tailored synthetic identity that holds the potential to elegantly precoat LNPs with an artificial protein corona. This endeavor seeks to create a corona with a precise composition strategically designed to preserve its inherent attributes once it is introduced into the human biological milieu. The complexity of this mission lies in engineering a corona that can navigate the intricacies of the human system, safeguarding its integrity and functionality, and ultimately culminating in a ground-breaking advancement in the realm of biomedical applications.

## Conclusion

As of May 20, 2023, COVID-19 is still classified as a pandemic by the WHO, highlighting the high desirability of technological advancements in this arena. The integration of the LNP-protein corona as a pioneering approach for vaccine development heralds a paradigm shift in the field, necessitating the acquisition and elucidation of foundational knowledge. This innovative concept holds immense promise, as it possesses the potential to revolutionize vaccine design and deployment strategies, thereby offering a universal platform that can be readily adapted to combat future global pandemics and outbreaks caused by previously unknown viruses for which human immunity is absent. By leveraging the principles and insights gleaned from this novel vaccine development strategy, we lay the foundation for a universal platform that can be readily adapted to tackle unforeseen viral threats and other human conditions, such as cancer and genetic diseases.
